# ﻿Three new species of the leafhopper genus *Mitjaevia* Dworakowska from Karst areas in Southwest China (Hemiptera, Cicadellidae, Typhlocybinae)

**DOI:** 10.3897/zookeys.1125.82258

**Published:** 2022-10-25

**Authors:** Guimei Luo, Jinqiu Wang, Yuehua Song

**Affiliations:** 1 School of Karst Science, Guizhou Normal University, Guizhou, Guiyang 550001, China Guizhou Normal University Guiyang China; 2 State Engineering Technology Institute for Karst Desertification Control, Guiyang 550001, China State Engineering Technology Institute for Karst Desertification Control Guiyang China

**Keywords:** Homoptera, morphology, new taxa, taxonomy

## Abstract

Three new species of the leafhopper genus *Mitjaevia*Dworakowska 1970, *M.bijiensis***sp. nov.**, *M.solitaria***sp. nov.**, and *M.salaxia***sp. nov.**, are described from the Karst region of Southwest China. Specimens studied were taken by sweep net. Morphological descriptions, depictions of habitus and illustrations of male terminalia are provided. A key and checklist to known species occurring in China are given.

## ﻿Introduction

The genus *Mitjaevia* was designated by Dworakowska in 1970 belonging to the tribe Erythroneurini of the subfamily Typhlocybinae (Hemiptera: Cicadellidae). Some members of the genus are known as important agricultural pests in the world. *Mitjaevia* Dworakowska share similar features with *Diomma* Motschulsky and *Kusala* Dworakowska such as the vertex and pronotum, usually with dark spots or stripes; the style with apex slender and curved; and connective with two strong lateral arms but can be distinguished by the combination of the following characters: (1) pygofer lobe with numerous microsetae or microtrichia near caudal area and basal lower angle without clusters of long macrosetae; (2) abdominal apodemes small, not extending beyond hind margin of 3^rd^ sternite; (3) forewing semitransparent, light brown or brown, often decorated with white or milky patches.

Later, Song et al. (2011) and Dmitriev (2020) supplemented features of dorsum, eyes, legs, abdomen, pygofer and pygofer dorsal appendage of *Mitjaevia* and also noted that forewings possess four apical cells, subgenital plates contains 2–4 basal macrochaetae and the aedeagus has a distal short apical processes, toothlike or absent. The species of this genus are mainly distributed in the Palearctic and Oriental regions. Until now, 19 species have been described, of which 11 species are in China (see Checklist). In the present paper, three new species of *Mitjaevia* from Guizhou Province are proposed to the Chinese fauna. A key to recognize all Chinese species is provided.

## ﻿Materials and methods

Specimens for this study were collected on roadside weeds in Karst (Guizhou Province, China) by sweep net. The morphological terms used in this study followed Dietrich (2005) and Song and Li (2013). A KEYENCE VHX-5000 digital microscope was used to take pictures of male habitus. The Olympus BX53 microscope was used to dissect the male genital and the Olympus SZX16 microscope was used for viewing and drawing the male genital. Body length was measured from the apex of vertex to the tip of forewing. All specimens examined were deposited in The School of Karst Science collection, at Guizhou Normal University (**GZNU**).

## ﻿Taxonomy

### ﻿ Mitjaevia

Taxon classificationAnimaliaHemipteraCicadellidae

Dworakowska

B3123E39-0554-552F-A4B5-4B4D275254B6

Mitjaevia Dworakowska, 1970: 763.

#### Type species.

*Erythroneuraamseli* Dlabola, 1961.

#### Type locality.

Afghanistan.

#### Diagnosis.

Yellow with brown markings; vertex usually with a pair of dark brown spots; pronotum with brown markings or completely dark; scutellum with dark basal triangles; forewing hyaline with several dark patches; abdomen ventrally and subgenital plates dark. Head distinctly narrower than pronotum.

#### Male genitalia.

Pygofer caudally rounded or angulate, weakly sclerotized caudally; dorsal pygofer appendage movably articulated, not extended beyond pygofer apex. Style distally slender with apex foot-like; preapical lobe large. Subgenital plates extended beyond pygofer, darkly pigmented, gradually curved dorsad, with three or more macrosetae of differing length present in middle. Aedeagus with shaft tubular, straight or curved dorsad in lateral view, with or without basal processes; preatrium developed. Connective Y- or M-shaped, with short stem and arms, central lobe well developed.

#### Distribution.

Palaearctic and Oriental regions.

##### ﻿Checklist of species of *Mitjaevia* from China

*M.aurantiaca* Mitjaev, 1969: 1044 (pl. 3, figs 1, 2); Song & Li, 2014: 111, (pl. 2.71, figs D-F; I, J, M, S).

*M.bifurcata* Luo, Song & Song, 2021: 3–6, fig. 2.

*M.diana* (Distant, 1918: 100, *Typhlocyba*); Dworakowska, 1970: 765 (lectotype designated by inference); Dworakowska, 1980: 179, figs 252–262; Song & Li, 2014: 112 (pl. 2.72, figs D-F; I, J, M).

*M.dworakowskae* Chen, Song & Webb, 2020: 34–39, figs 28–41.

*M.korolevskayae* Dworakowska, 1979: 44–45, figs 349–358; Song & Li, 2014: 113 (pl. 2.73, figs D-F; I, J, M).

*M.nanaoensis* Chiang & Knight, 1990: 223, fig. 18.

*M.protuberanta* Song, Li & Xiong, 2011: 27–29, figs 1–10.

*M.bijiensis* sp. nov.

*M.ramosa* Luo, Song & Song, 2021: 6–8, fig. 5.

*M.salixia* sp. nov.

*M.shibingensis* Chen, Song & Webb, 2020: 34, fig. 15–27.

*M.solitaria* sp. nov.

*M.tappana* Chiang & Knight, 1990: 225, fig. 19.

*M.wangwushana* Song, Li & Xiong, 2011: 29–30, figs 11–19.

### ﻿Key to species of *Mitjaevia* from China (males)

**Table:** 

1	Aedeagus with single or paired processes	**2**
–	Aedeagus without processes	**10**
2	Aedeagal shaft with paired processes	**3**
–	Aedeagal shaft with single process (Fig. 25)	***M.solitaria* sp. nov.**
3	Aedeagus with apical, subapical or basal processes, but not dorsally	**4**
–	Aedeagus only with dorsal processes	***M.bifurcata***
4	Aedeagal shaft with apical process	**5**
–	Aedeagal shaft without apical process	**7**
5	Aedeagal shaft with unbifurcated apical process (Fig. 17)	***M.bijiensis* sp. nov.**
–	Aedeagal shaft with bifurcated apical process	**6**
6	Aedeagal shaft with pair of asymmetric bifurcated short processes at apex	***M.ramosa***
–	Aedeagal shaft with pair of symmetrical bifurcated short processes at apex	***M.diana***
7	Aedeagal shaft with paired processes subapically	**8**
–	Aedeagal shaft without paired processes subapically	**9**
8	Aedeagal shaft with pair of small, triangle-like processes subapically	***M.protuberanta***
–	Aedeagal shaft with pair of long, curved processes subapically	***M.wangwushana***
9	Aedeagal shaft with finger-like basal processes ventrally	***M.aurantiaca***
–	Aedeagal shaft with lamellate-like basal processes ventrally (Fig. 33)	***M.salaxia* sp. nov.**
10	Preatrium of aedeagus long in lateral view	**11**
–	Preatrium of aedeagus short in lateral view	***M.tappana***
11	Aedeagal shaft cylindrical, evenly tapered from base to apex	**12**
–	Aedeagal shaft laterally compressed, abruptly tapered from subapically to apex	**13**
12	Aedeagus dorsal apodeme visible in lateral view	***M.korolevskayae***
–	Aedeagus dorsal apodeme absent or vestigial in lateral view	***M.nanaoensis***
13	Aedeagal shaft with rounded apex in lateral view; preatrium expanded in ventral view	***M.shibingensis***
–	Aedeagal shaft with acute apex in lateral view; preatrium narrow in ventral view	***M.dworakowskae***

### ﻿Descriptions

#### ﻿ Mitjaevia bijiensis sp. nov.

Taxon classificationAnimaliaHemipteraCicadellidae

0F83B222-6FF1-5EEC-BD44-F2E89A0FA7D4

https://zoobank.org/77C7830A-4369-4A7E-96C6-D099579FDC07

Figs 1–5 , 16–23

##### Material examined.

***Holotype***: ♂, China: Guizhou Province, Bijie, 6.VI.2021, coll. Jia Jiang and Ni Zhang. ***Paratypes***: 1 ♀, same data as holotype.

##### Diagnosis.

The new species can be distinguished from other species by the aedeagal shaft long with two apical processes; preatrium of aedeagus with two atrial processes. The head and pronotum yellow. Pygofer dorsal appendage tapered to apex and bent back into a hook shape. Style apex slightly expanded, underpart straight and thick. Connective with large central lobe.

##### Description.

**Figures 1–15.**
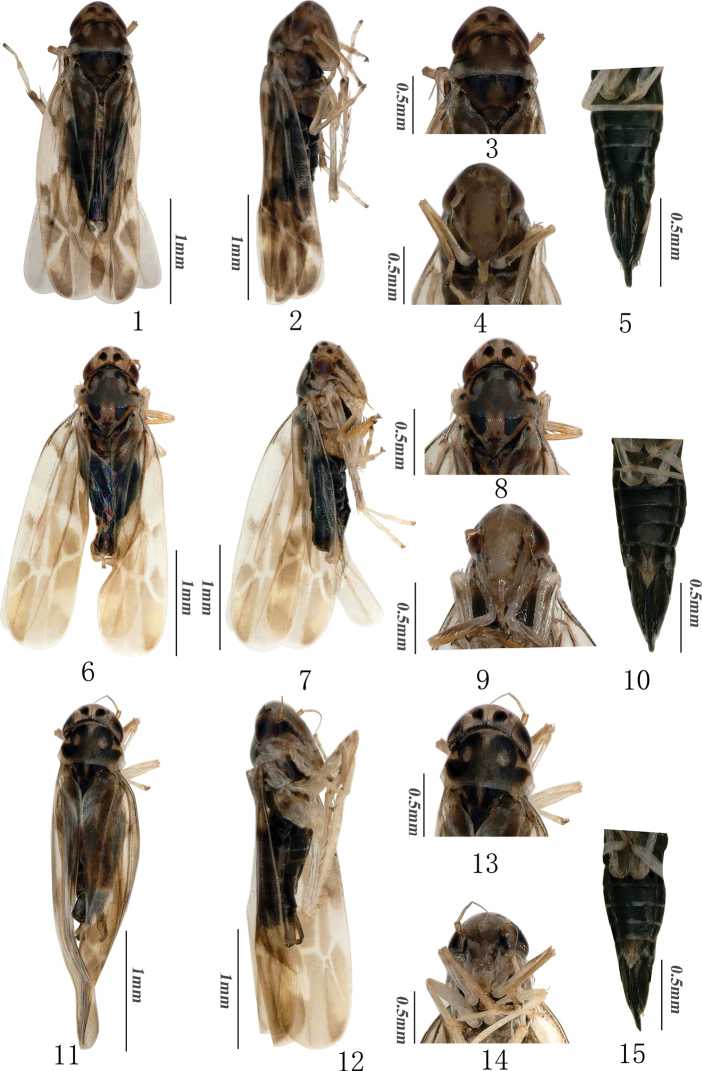
External morphology of *Mitjaevia* species **1–5***Mitjaeviabijiensis* sp. nov. **1** habitus, dorsal view **2** habitus, lateral view **3** head and thorax, dorsal view **4** face **5** female thorax and abdomen, ventral view **6–10***Mitjaeviasolitaria* sp. nov. **6** habitus, dorsal view **7** habitus, lateral view **8** head and thorax, dorsal view **9** face **10** female thorax and abdomen ventral view **11–15***Mitjaeviasalaxia* sp. nov. **11** habitus, dorsal view **12** habitus, lateral view **13** head and thorax, dorsal view **14** face **15** female thorax and abdomen, ventral view.

Head and thorax yellow marked with brown; vertex with a pair of dark brown spots (Figs 1–4). Pronotum yellow, with irregularly dark brown patch medially (Figs 1, 3). Scutellum yellow, transverse impression distinct. Face brownish yellow (Fig. 1); anteclypeus with black patches at sides basally (Fig. 4). Forewing brownish, with large milky white or whitish patches.

Abdominal apodemes long, exceeding posterior margin of 3^rd^ sternite (Fig. 23).

**Figures 16–23.**
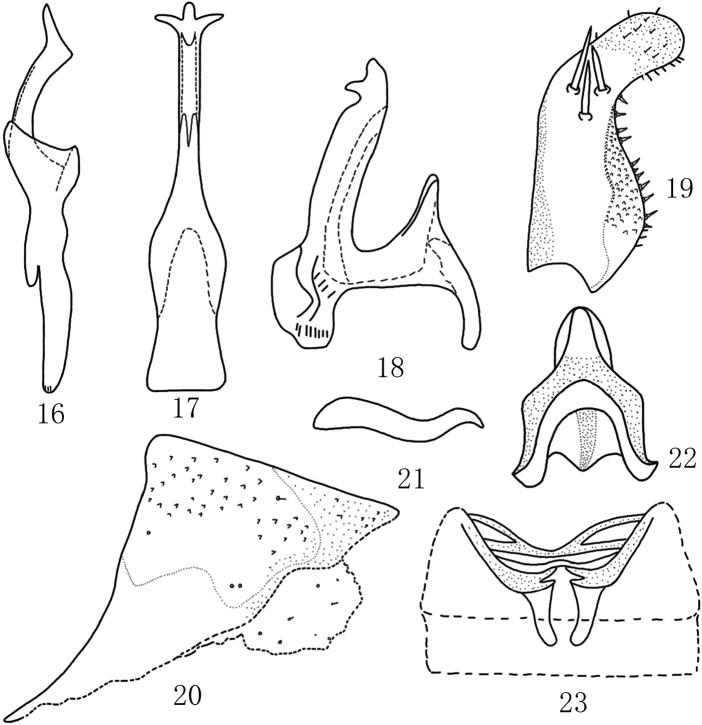
*Mitjaeviabijiensis* sp. nov. **16** style **17** aedeagus, ventral view **18** aedeagus, lateral view **19** subgenital plate **20** male pygofer, lateral view **21** pygofer dorsal appendage, lateral view **22** connective **23** abdominal apodemes.

***Male genitalia*.** Pygofer lobe broad, with sparse fine setae on lateral surface (Fig. 20). Pygofer dorsal appendage slightly expanded at base, bends up and down and tapers gradually (Fig. 21). Subgenital plate with three macrosetae of different lengths medially (Fig. 19). Style long and strong; preapical lobe large (Fig. 16). Aedeagal shaft long, with pair of short bifurcate apical processes; preatrium also with two atrial processes (Figs 17, 18). Connective M-shaped, with distinct stem and central lobe (Fig. 22).

***Body length* (including wings).** ♂, 2.9–3.0 mm, ♀, 2.8–2.9 mm.

##### Remarks.

The new species is similar to *Mitjaeviadiana* (Distant, 1918) but can be distinguished by the aedeagal shaft with two apical processes and preatrium of aedeagus with two atrial processes; the style apex slightly expanded, underpart straight and thick; the connective with large central lobe.

##### Etymology.

The new species is named after its type locality Bijie City in China.

#### ﻿ Mitjaevia solitaria sp. nov.

Taxon classificationAnimaliaHemipteraCicadellidae

EB22731B-762A-51B9-9A18-0D45549F5AC9

https://zoobank.org/29D48CC0-BE39-4F3D-983C-53FE2CD472B3

Figs 6–10 , 24–31

##### Material examined.

***Holotype***: ♂, China: Guizhou Province, Bijie, 6.VI.2021, coll. Jia Jiang and Ni Zhang. ***Paratypes***: 18 ♂♂, 3 ♀♀, same data as holotype.

##### Diagnosis.

The new species can be distinguished from other species by the aedeagal shaft with only one thick finger-like process at base. Pygofer dorsal appendage not extended beyond hind margin of pygofer. Style strong; preapical lobe obvious. Subgenital plate wide and short with one hook-like process on apex.

##### Description.

Vertex yellowish (Fig. 6). Pronotum mostly grayish black (Figs 6, 8). Scutellum yellowish, transverse impression distinct (Fig. 6). Face brownish yellow; anteclypeus brownish except lighter base (Fig. 9). Forewing brownish, with large milky white or whitish patches.

Abdominal apodemes small, located in 3^rd^ sternite (Fig. 31).

**Figures 24–31.**
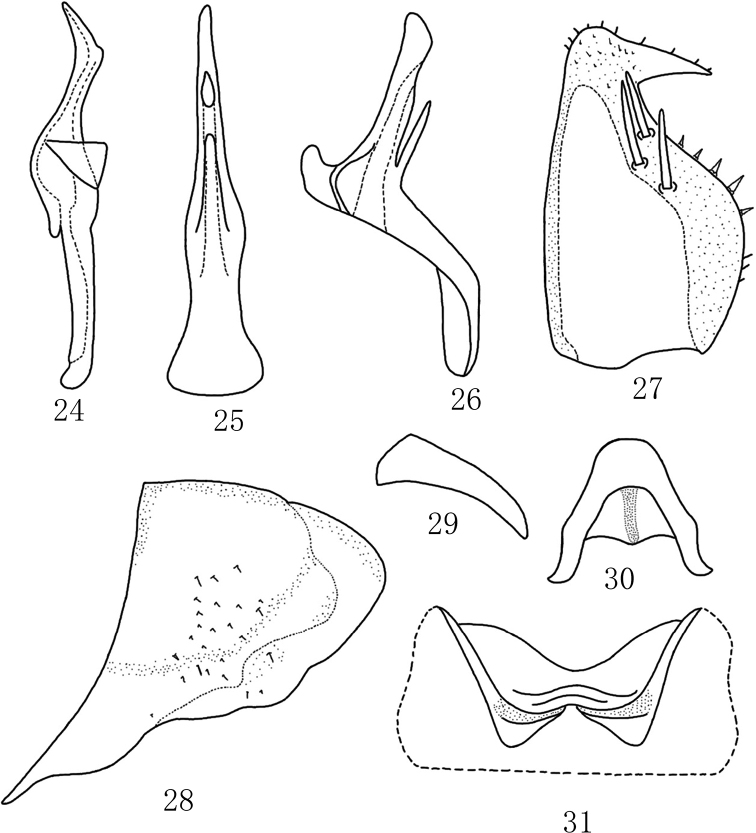
*Mitjaeviasolitaria* sp. nov. **24** style **25** aedeagus, ventral view **26** aedeagus, lateral view **27** subgenital plate **28** male pygofer, lateral view **29** pygofer dorsal appendage, lateral view **30** connective **31** abdominal apodemes.

***Male genitalia*.** Pygofer dorsal appendage weakly expanded at base, not extended beyond hind margin of pygofer (Fig. 29). Pygofer lobe broad, with fine setae scattered outer lateral surface (Fig. 28). Subgenital plate wide and short, with three macrosetae on lateral surface, and one hook-like process on apex (Fig. 27). Style strong; preapical lobe obvious (Fig. 24). Aedeagal shaft as long as or little shorter than that of preatrium, with one finger-like process basally; dorsal apodeme small (Figs 25, 26). Connective Y-shaped; arms and central lobe slender (Fig. 30).

***Body length* (including wings).** ♂, 3.1–3.2 mm, ♀, 3.0–3.2 mm.

##### Remarks.

The new species is similar to *M.aurantiaca* (Mitjaev, 1969), but can be distinguished by the aedeagal shaft with only one thick finger-like process at base; the style is stronger and the subgenital plate is shorter and wider.

##### Etymology.

The new species is named from the Latin word *solitarius*, referring to the aedeagal shaft with only one processes at the base.

#### ﻿ Mitjaevia salaxia sp. nov.

Taxon classificationAnimaliaHemipteraCicadellidae

C961304C-9D8B-510D-BB42-9A090C2277CB

https://zoobank.org/278419B3-C800-4929-A7AD-6B9BBBFAA056

Figs 11–15 , 32–39

##### Material examined.

***Holotype***: ♂, China: Guizhou Province, Bijie, 5.VI.2021, coll. Jia Jiang and Ni Zhang. ***Paratypes***: 1 ♂♂, 6 ♀♀, same data as holotype.

##### Diagnosis.

The new species can be identified by the two pairs of abdominal apodemes and the aedeagal shaft with lamellate-like processed at base. Style apex long and slender. Subgenital plate long, expanded near caudal.

##### Description.

Vertex pale yellow (Fig. 11). Pronotum yellowish, with symmetrical brownish black impressed patches medially (Figs 11, 13). Scutellum (Fig. 11) yellow, with basal triangles black and area under transverse impression black too. Face brownish gray; anteclypeus with central area brownish (Fig. 14). Forewing brownish, with large milky white or whitish patches.

Second abdominal apodemes and third abdominal apodemes small and short, lamellate, not exceeded 3^rd^ sternite (Fig. 39).

**Figures 32–39.**
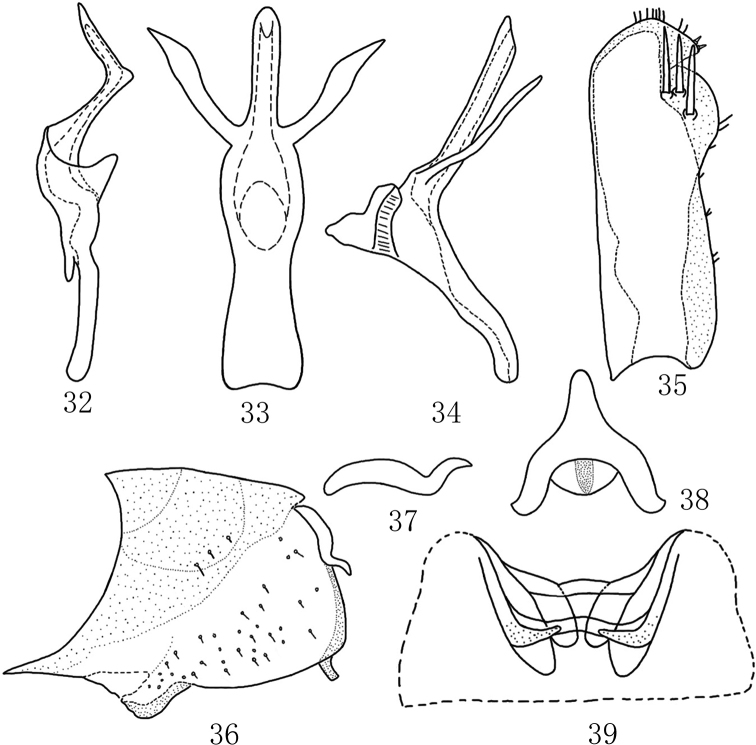
*Mitjaeviasalaxia* sp. nov. **32** style **33** aedeagus, ventral view **34** aedeagus, lateral view **35** subgenital plate **36** male pygofer, lateral view **37** pygofer dorsal appendage, lateral view **38** connective **39** abdominal apodemes.

***Male genitalia*.** Pygofer dorsal appendage simple, curved upward in lateral view, hook-like apically (Fig. 37). Pygofer lobe broad, with many long fine setae on lateral surface, with a small process at caudal edge ventrally (Fig. 36). Subgenital plate long, with three macrosetae near apex (Fig. 35). Style apex long and slender; preapical lobe large (Fig. 32). Aedeagal shaft slender, with pair of long processes arising from base (Figs 33, 34). Connective Y-shaped; stem slender; arms and central lobe well developed (Fig. 38).

***Body length* (including wings).** ♂, 3.0–3.1 mm, ♀, 2.9–3.0 mm.

##### Remarks.

The new species is similar to *M.protuberanta* Song, Li & Xiong, 2011, but differs in having the “lamellate” processed arising from base of aedeagal shaft and not branched at apex; with two pairs of abdominal apodemes.

##### Etymology.

The new species is named after its type locality Salaxi town.

## ﻿Discussion

In recent years, most research on Chinese Erythroneurine leafhoppers has been intensified and focused to enrich the taxonomic knowledge of this tribe and taxonomists have paid attention to documenting taxa using efficient descriptions, high-quality drawings, and photographs. The Guizhou province is located on the eastern slope of the Yunnan-Guizhou Plateau in southwestern China and has a particularly subtropical humid monsoon climate with four distinct seasons, abundant rainfall, seasonal temperature variations and high vegetation coverage, which is conducive to the survival and reproduction of leafhoppers. Since establishment of this genus, 19 species of *Mitjaevia* have been described worldwide, and more than half of valid species were found in China. Here, a comparison revealed that three new species shared similarities with already known species but differences were found, for example, the aedeagal shaft of *M.bijiensis* sp. nov. and *M.diana* have apical processes, but *M.bijiensis* sp. nov. dispose processes arising from preatrium of aedeagus, while *M.diana* shows another processes at base of aedeagal shaft. *Mitjaevia.solitaria* sp. nov. and *M.aurantiaca* also have a single process at base of aedeagal shaft, not paired. Moreover, the subgenital plate is short and wide but in latter species it is long or thin. *Mitjaevia.salaxia* sp. nov. is similar to *M.protuberanta* but differs in having lamellate-like processes arising from the base of aedeagal shaft and not branched at apex.

## Supplementary Material

XML Treatment for Mitjaevia

XML Treatment for Mitjaevia bijiensis

XML Treatment for Mitjaevia solitaria

XML Treatment for Mitjaevia salaxia

## References

[B1] Chen XX Song YH Webb MD (2020) Two new species of the leafhopper genus Mitjaevia Dworakowska from China (Hemiptera, Cicadellidae, Typhlocybinae). ZooKeys 964(1): 31–40. 10.3897/zookeys.964.48655 32939146 PMC7471134

[B2] Chiang CC Knight WJ (1990) Studies on taiwanese Typhlocybinae (Homoptera: Cicadellidae) (IV) tribe Erythroneurini. Bulletin of the National Museum of Natural Science 2: 191–255.

[B3] Dietrich CH (2005) Keys to the families of Cicadomorpha and subfamilies and tribes of Cicadellidae (Hemiptera: Auchenorrhyncha). The Florida Entomologist 88(4): 502–517. 10.1653/0015-4040(2005)88[502:KTTFOC]2.0.CO;2

[B4] Dlabola J (1961) Zikaden von Zentralasien, Dagestan und Transkaukasien (Homopt. Auchenorrhyncha). Acta Entomologica Musei Nationalis Pragae 34: 241–358.

[B5] Dmitriev D (2020) An online interactive key and searchable database of Auchenorrhyncha (Hemiptera). http://dmitriev.speciesfile.org/

[B6] Dworakowska I (1970) On the genera *Asianidia* Zachv. and *Singapora* Mahm. with the description of two new genera (Auchenorrhyncha, Cicadellidae, Typhlocybinae). Bulletin de l`Académie Polonaise des Sciences, Série des Sciences Biologiques 18(12): 759–765.

[B7] Dworakowska I (1979) On some Erythroneurini from Vietnam (Typhlocybinae, Cicadellidae). Annotationes Zoologicae et Botanivae 131: 1–50.

[B8] Dworakowska I (1980) On some Typhlocybinae from India (Homoptera, Auchenorrhyncha, Cicadellidae). Entomologische Abhandlungen Staatliches Museum Für Tierkunde in Dresden 43(8): 151–201. 10.1515/9783112653227-010

[B9] Luo GM Song QF Song YH (2021) Two new species of the leafhopper genus *Mitjaevia* Dworakowska from China (Hemiptera, Cicadellidae, Typhlocybinae). Biodiversity Data Journal 9: e72420. 10.3897/BDJ.9.e72420 PMC8520034 34720638

[B10] Mitjaev ID (1969) New species of leaf-hoppers (Homoptera, Cicadinea) from Tien Shan and Karatau. Zoologicheskii Zhurnal 48(7): 1041–1047.

[B11] Song YH Li ZZ (2013) Two new species of *Empoascanara* Distant (Hemiptera: Cicadellidae: Typhlocybinae) from Yunnan Province, China. Zootaxa 3637(1): 089–093. 10.11646/zootaxa.3637.1.11 26046182

[B12] Song YH Li ZZ (2014) Erythroneurini and Zyginellini from China (Hemiptera: Cicadellidae: Typhlocybinae). Science and Technology Publishing House, Guiyang, 12–209.

[B13] Song YH Li ZZ Xiong KN (2011) Two new species of the genus Mitjaevia Dworakowska from China (Hemiptera: Cicadellidae: Typhlocybinae). Zootaxa 2805(1): 26–30. 10.11646/zootaxa.2805.1.2

